# Lloyd’s Maps

**Published:** 1862-11

**Authors:** 


*Lloyd's Maps.*—We have received Lloyd's Map of Virginia, Lloyd's Map of the United States, or rather of the *un*-United States, and also his Map of the Mississippi from St. Louis to the Gulf of Mexico, showing every bend in the river, every island, sand bar, landing, town, city, bluff, sugar and cotton plantation, and the name of the owners. It shows also the counties bordering on the river on each side ten miles back from the river, with the towns, villages and post offices, and the roads leading to them. Also all the streams emptying into the Mississippi. It is engraved in six sections of five feet each, in length, and printed upon linen paper, so as to be conveniently folded and sent by mail like a newspaper. One of the most remarkable features of all, it is sold for one dollar per copy.

Lloyd's Map of Virginia is also the best possible map of the war, and we find that it is the standard authority in the army. The locations of the towns made historic by the recent battles and all other objects of interest in the geography of that State are represented, together with the District of Columbia, Maryland, Delaware, and a part of Pennsylvania and New Jersey.

The Map of the United States is a most magnificent map of all the States; the counties distinguished by different colors. Towns, railroads, rivers, mountains, distances and indeed everything which can go to make up the perfection of a map will be found included. It is on strong, fine paper, four feet in length and width, and in every respect a very complete map. What seems to us the most remarkable of all is, its price. It is sold for fifty cents, and all the other maps by Mr. Lloyd in about the same proportion. That it can be made so complete is quite remarkable; that it is so cheap is to us inexplicable.

These maps may be obtained of the publishers through the Post Office, by addressing J. T. Lloyd, 164 Broadway, N. Y.

